# A Comparison of Theory-Based and Experimentally Determined Myocardial Signal Intensity Correction Methods in First-Pass Perfusion Magnetic Resonance Imaging

**DOI:** 10.1155/2015/843741

**Published:** 2015-09-30

**Authors:** Jacob U. Fluckiger, Brandon C. Benefield, Lara Bakhos, Kathleen R. Harris, Daniel C. Lee

**Affiliations:** ^1^Department of Radiology, Northwestern University Feinberg School of Medicine, Chicago, IL 60611, USA; ^2^Feinberg Cardiovascular Research Institute, Northwestern University Feinberg School of Medicine, Chicago, IL 60611, USA; ^3^Division of Cardiology, Department of Medicine, Loyola University Chicago Stritch School of Medicine, Maywood, IL 60153, USA; ^4^Division of Cardiology, Department of Medicine, Northwestern University Feinberg School of Medicine, Chicago, IL 60611, USA

## Abstract

*Objectives.* To evaluate the impact of correcting myocardial signal saturation on the accuracy of absolute myocardial blood flow (MBF) measurements. *Materials and Methods.* We performed 15 dual bolus first-pass perfusion studies in 7 dogs during global coronary vasodilation and variable degrees of coronary artery stenosis. We compared microsphere MBF to MBF calculated from uncorrected and corrected MRI signal. Four correction methods were tested, two theoretical methods (Th1 and Th2) and two empirical methods (Em1 and Em2). *Results.* The correlations with microsphere MBF (*n* = 90 segments) were: uncorrected (*y* = 0.47*x* + 1.1, *r* = 0.70), Th1 (*y* = 0.53*x* + 1.0, *r* = 0.71), Th2 (*y* = 0.62*x* + 0.86, *r* = 0.73), Em1 (*y* = 0.82*x* + 0.86, *r* = 0.77), and Em2 (*y* = 0.72*x* + 0.84, *r* = 0.75). All corrected methods were not significantly different from microspheres, while uncorrected MBF values were significantly lower. For the top 50% of microsphere MBF values, flows were significantly underestimated by uncorrected SI (31%), Th1 (25%), and Th2 (19%), while Em1 (1%), and Em2 (9%) were similar to microsphere MBF. *Conclusions.* Myocardial signal saturation should be corrected prior to flow modeling to avoid underestimation of MBF by MR perfusion imaging.

## 1. Introduction

Vasodilated first-pass magnetic resonance myocardial perfusion imaging has been shown to detect significant coronary artery stenoses [1–5] and predict prognosis [6, 7] in patients with coronary artery disease. Typical perfusion acquisitions involve the serial acquisition of *T*_1_-weighted images before, during, and immediately following intravenous injection of a gadolinium-based contrast agent (CA). Most studies are assessed either visually or semiquantitatively to identify areas with poor contrast enhancement relative to other regions of myocardium. Absolute myocardial blood flow in mL/min/g (MBF) can be derived from the time courses of the concentration of CA in the myocardium and either the left ventricular (LV) blood pool or ascending aorta [8–11]. In experimental studies, MBF correlated better with microspheres than semiquantitative methods [12], and in patients with coronary artery disease qualitative assessment alone underestimated the extent or even missed the presence of ischemia compared to calculation of perfusion reserve from MBF [13]. However, due to the nonlinear relationship between signal intensity (SI) and CA concentration, the imaging signal must be corrected prior to MBF calculation to avoid systematic error.

Several methods have been developed to avoid signal saturation in the relatively high concentrations of CA found in the LV blood pool. These techniques include dual bolus injection of CA [12–15], dual echo or dual delay time imaging [16], multiple subset reconstructions with multiple delay times [10], and blind estimation of the LV blood pool signal [8, 17]. Each of these techniques has been shown to return an SI time course in the blood pool that is linearly related to the concentration of CA, which then can be used in quantitative perfusion modeling. Although CA concentrations achieved in the myocardium are significantly lower than those seen in the LV blood pool, significant myocardial signal saturation has been demonstrated in first-pass perfusion studies using standard clinical CA doses up to 0.1 mmol/kg [18, 19]. Reduction of CA dose can mitigate signal saturation, but for the diagnosis of coronary artery disease use of higher CA doses (0.1–0.15 mmol/kg) has been shown to improve diagnostic accuracy over a lower dose (0.05 mmol/kg) [20]. To avoid underestimation of myocardial blood flow, standard quantitative algorithms cannot be applied to the time-signal intensity data without also correcting the myocardial tissue signal.

By understanding the relationship between myocardial signal intensity and CA concentration, myocardial signal saturation can be corrected prior to the application of quantitative perfusion models. Two empirically derived [21] and two theory-based [19, 22] correction algorithms have been proposed to derive the signal intensity-CA concentration relationship. The purpose of this work is to compare the accuracy of myocardial blood flow estimates from each of these correction methods in a canine model of vasodilator stress with varying degrees of coronary artery stenosis. A dual bolus protocol was implemented to obtain the corrected LV blood pool signal and quantitative modeling was applied to the uncorrected myocardial signal, as well as signal corrected with both theory-based and empirically determined corrections. Absolute MBF values calculated from injected microspheres served as the gold standard for each of the perfusion quantification methods. The successful validation of these techniques would constitute an important step in improving the accuracy of absolute MBF measurements by first-pass perfusion magnetic resonance imaging. Furthermore, because these techniques can be applied retrospectively, absolute MBF can be measured from routine clinical perfusion studies using standard CA doses.

## 2. Materials and Methods

### 2.1. First-Pass Perfusion Imaging

A total of fifteen studies were conducted on seven dogs in accordance with and after approval by our institution's animal care and use committee. Each animal was chronically instrumented with an external hydraulic occluder and cuff-type Doppler flowmeter around the left circumflex or left anterior descending coronary artery as described previously [23]. Left atrial, right atrial, and aortic catheters were placed for the administration of fluorescent microspheres, phenylephrine, and withdrawal of reference blood samples, respectively. Each animal was allowed to recover for at least 48 hours between imaging studies. All perfusion studies were performed under maximal adenosine vasodilation. The adenosine infusion rate for each dog (140–420 mcg/kg/min) was that which produced the greatest increase in Doppler flow on a preliminary study. Except during reference blood withdrawals, the aortic catheter was used for continuous invasive blood pressure monitoring, and phenylephrine (40–80 mcg/min) was given to maintain mean arterial pressure > 60 mmHg. Different levels of coronary stenosis were achieved by varying the inflation level of the coronary occluder under Doppler flowmeter guidance for each study. During image acquisition ventilation was suspended to eliminate respiratory motion artifacts.

All perfusion images were acquired with a 1.5T scanner (Siemens Medical Systems, Erlangen, Germany) with a saturation recovery, Cartesian, and turboFLASH sequence (TR/TE = 2.21/1.39 ms, saturation recovery time = 100 ms, flip angle = 12°, slice thickness = 8 mm, in-plane resolution = 1.79 mm, and acquisition matrix = 192 × 74). GRAPPA acceleration with an acceleration factor of 2 was used and no fat saturation was applied. Two or three short axis slices were scanned depending on the animal's heart rate at the time of imaging and the basal and mid-ventricular slices were selected for further analysis. Using a dual bolus protocol, two equal volume doses of gadopentetate dimeglumine (Magnevist, Bayer Healthcare, Whippany, NJ; 0.005 mmol/kg and 0.05 mmol/kg) were injected using separate power injectors (Medrad Inc., Indianola, PA) at a constant rate of 4 mL/s followed by a 12 mL saline flush injected at 4 mL/s. Immediately following the high-dose contrast injection of each study, approximately 3 × 10^6^ microspheres (FluoSpheres Blood Flow Determination Color Kit #2, 15 *μ*m, Invitrogen, Eugene, Oregon) were injected via catheter into the left atrium with simultaneous reference blood withdrawal from the aortic catheter. Microspheres with multiple unique fluorescence spectra enabled multiple imaging studies to be carried out in each animal.

### 2.2. MRI Signal Intensity Corrections

Two of the signal intensity correction methods compared here are based on magnetization modeling and are referred to in the text as theoretical method 1 (Th1) and theoretical method 2 (Th2). Briefly, Th1, described in detail by Cernicanu and Axel [22], calculates the time-evolution of the magnetization through repeated application of the Bloch equations [24] to arrive at a functional representation of the magnetization signal in terms of the imaging parameters and the longitudinal relaxation time (*T*_1_) of the tissue. The signal is normalized by a precontrast, proton density weighted image to eliminate coil sensitivity weighting in the image. This method assumes that *T*_2_ effects on the signal are negligible and that the magnetization is completely saturated at the beginning of each image acquisition. Following image acquisition, the normalized signal is converted into *T*_1_ values, which can then subsequently be converted to CA concentration by

(1) $$\begin{array}{c} \frac{1}{T_{1}}=\frac{1}{T_{1,0}}+r_{1}\left[\;CA\;\right], \end{array}$$

where *T*_1,0_ refers to the precontrast *T*_1_ value and *r*_1_ is the relaxivity of CA. In our implementation, we modify the method described in [22] by replacing the echo planar imaging (EPI) signal equation with the appropriate signal equation for fast low-angle shot (turboFLASH) imaging. In addition, rather than normalizing the dynamic image series by a proton density weighted scan, we instead normalize by the mean precontrast signal prior to contrast injection; we return to this point in Discussion. As in [22], the two steps in Th1 (conversion from signal to *T*_1_ and from *T*_1_ to CA) are combined into a single calibration curve relating SI to CA which is used as a look-up table to decrease total processing time.

Th2, described in detail by Hsu et al. [19], is based on the theoretical work of Sekihara [25]. This method simulates the effects of repeated radio frequency (RF) pulses on a set of spin isochromats. The net magnetization vector of the spin isochromats following both the saturation and readout RF pulses is calculated for a given set of imaging parameters and pulse sequence design. As with Th1, Th2 normalizes SI by a precontrast, proton density weighted image and creates a look-up table relating SI and different concentrations of CA which can be used to correct the acquired signal. Again, in our implementation we modify the method described in [19] by substituting a turboFLASH acquisition into the isochromat simulation and normalizing the image series by the mean precontrast signal prior to contrast injection.

The third correction method tested here, denoted as empirical method 1 (Em1), uses an experimentally determined relationship between SI and CA concentration to correct for myocardial signal saturation. In this method, described by Lee et al. in [21], the relationship between SI and CA concentration was derived by alternately measuring SI and *T*_1_ during the constant, slow infusion of gadopentetate dimeglumine (0.33 mmol/min) in a dog. SI was measured using a standard perfusion sequence (described above), *T*_1_ mapping was performed with a modified Look-Locker (MOLLI) technique [26], and CA concentration was calculated from MOLLI *T*_1_ values using (1). Baseline-corrected myocardial SI and CA concentration were plotted as functions of infusion time to generate a SI-CA concentration response curve. The SI from the perfusion imaging is normalized by the precontrast signal intensity, and the SI-CA concentration curve was used as a lookup table to correct signal saturation prior to absolute MBF calculation.

The final correction method used here, denoted as empirical method 2 (Em2), is based on the data collected in Em1. A fourth-order polynomial function was fit to the relative SI versus CA concentration data in a least squares sense. This heuristic model was selected based on qualitative observation of the best fit to the data. The resulting smoothed curve was used to generate a lookup table as with the Em1 method.

### 2.3. Data Analysis

Following acquisition the basal and mid-axis slice from each imaging session were selected for analysis and the myocardium was manually segmented by an experienced user. Each slice was divided into six equiangular regions, and the mean SI time course was calculated for each region. Relative signal enhancement images were generated by subtracting the mean baseline (precontrast) SI. These enhancement curves were then normalized by the precontrast signal as described above.

MBF values were compared from these SI curves without any correction and after correction by the Th1, Th2, Em1, and Em2 methods. All MBF calculation was performed using custom software developed in MATLAB (The MathWorks Inc., Natick, MA). Individual arterial input functions were taken from each low-dose injection dataset by calculating the mean signal intensity in a region of interest drawn in the LV blood pool and scaling the signal by 10 to correct for the difference in dose. The Tofts-Kety two-compartment model [27] was used to calculate *K* ^trans^ for each corrected and uncorrected SI curve using the individual input function. MBF was calculated from *K* ^trans^ after correcting for the extraction fraction of gadolinium. An extraction fraction of 0.46 was assumed for regions with MBF less than or equal to 2.0 mL/min/g and 0.32 for higher MBF regions [28].

After the completion of all imaging sessions, the animals were euthanized with an overdose of pentobarbital. Each heart was then fixed in formalin. The 8 mm slices, corresponding to the slices from the data analysis described above, were sectioned into six equiangular segments. Concentrations of fluorescent microspheres in each segment were quantified fluorometrically [29] and expressed on a per gram basis. Flow results from the microsphere analysis were compared with those from each of the signal saturation correction methods. Each pair of flow results was plotted against each other and the linear correlations were calculated. Bland-Altman mean-difference plots [30] were also generated for each pair of flow results to analyze the agreement between flow values. Generalized estimating equations [12] were used in the analysis to account for multiple data points being included from each imaging experiment. A one-way analysis of variance test was used to determine if any of the groups were significantly different at the 5% confidence level. Bonferroni's correction was used to adjust for multiple comparisons.

## 3. Results

**Figure 1:**
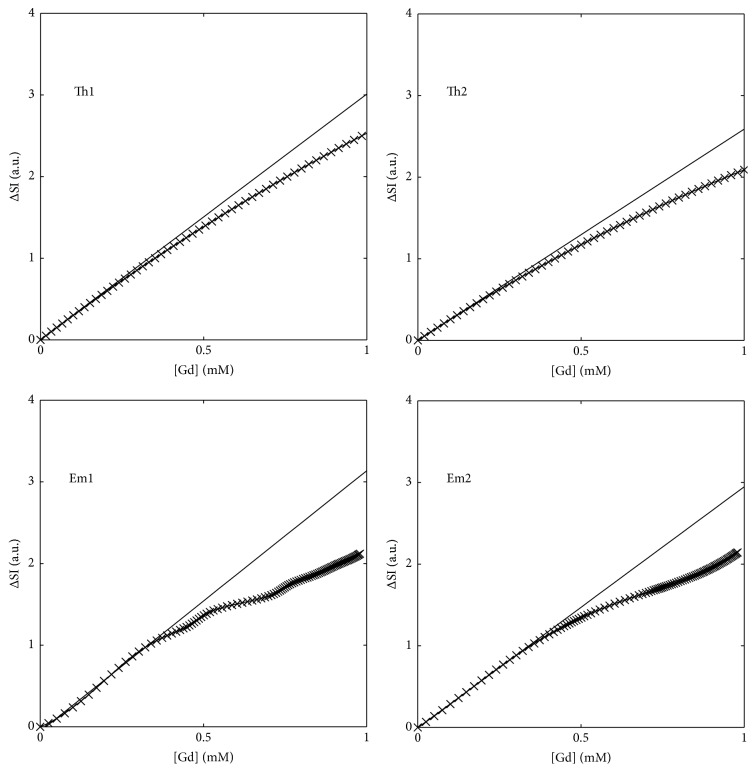
Panels showing the relationship between contrast agent concentration and relative signal enhancement for four signal correction methods. In each panel, the straight line represents the unsaturated (linear) signal expected with an increase in concentration.

**Figure 2:**
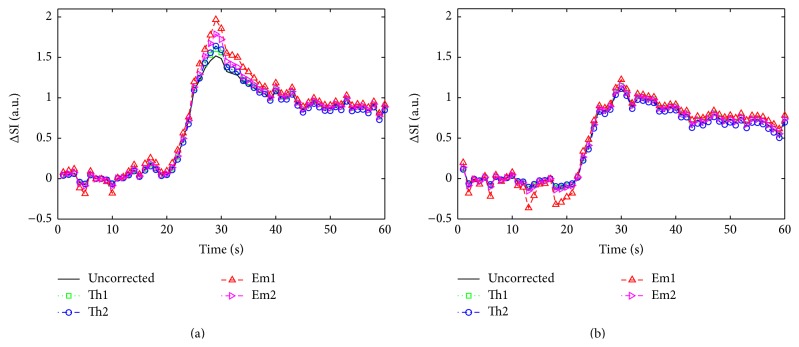
Representative tissue enhancement curves from a region of high flow (a) and low flow (b) within the myocardium of a single canine. Each panel displays the uncorrected relative enhancement, as well as the signal corrected by each of the methods investigated here.

Figure 1 displays the nonlinear relationship between CA concentration and relative signal enhancement for each of the correction methods used here. In each case the reference linear relationship between signal and concentration is also shown. The magnitude of the signal saturation at a CA concentration of 0.5 mmol/L was 10.0%, 9.4%, 11.4%, and 8.5% for Th1, Th2, Em1, and Em2, respectively. At a CA concentration of 1.0 mmol/L the signal saturation increased to 15.3%, 19.2%, 32.4%, and 27.3%, respectively. Representative relative tissue enhancement curves from two myocardial segments from a single imaging session are shown in Figure 2. The left panel shows a curve from a region with moderate to high signal enhancement. Correction of the signal intensity shows peak saturation of the signal to be 10.7%, 13.1%, 29.6%, and 15.1% for the Th1, Th2, Em1, and Em2 methods, respectively. The right panel displays a curve with low to moderate signal enhancement. The peak signal saturation for this curve was 3.5%, 0.1%, 10.0%, and 3.6%, respectively.

**Figure 3:**
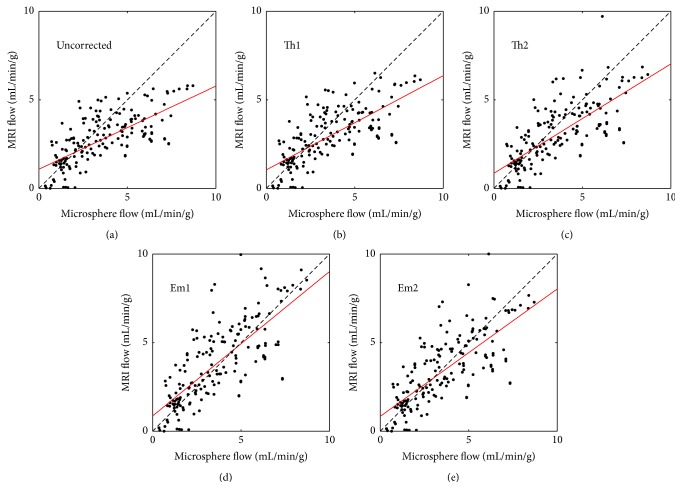
Scatter plots showing the relationship between the MBF calculated with fluorescent microspheres and MR perfusion imaging for uncorrected MR (a), Th1 (b), Th2 (c), Em1 (d), and Em2 (e). In each panel the line of unity (dashed, black) and line of best fit (solid, red) are shown for reference.

**Figure 4:**
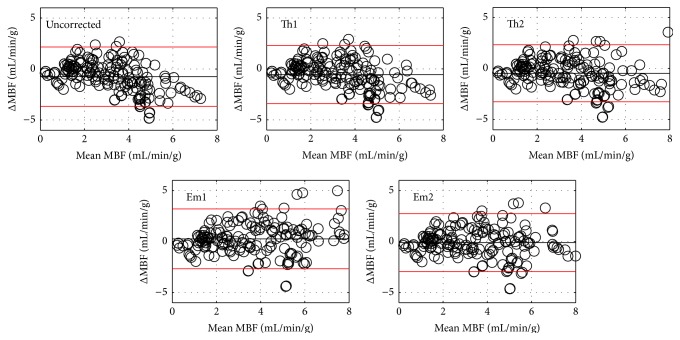
Bland-Altman plots for the data plotted in Figure 3. In each plot the mean difference is shown as a solid black line and the red lines represent the 95% confidence interval.

**Figure 5:**
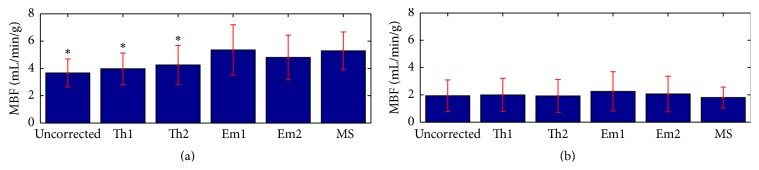
Mean (± one standard deviation) MBF values for the uncorrected and corrected MRI values, along with the microsphere results (MS). (a) represents data from the upper 50% of the flow values and (b) corresponds to the lower 50%. Results significantly different from the microsphere values are labeled (*∗*).

Summary results from all fifteen perfusion imaging studies are shown in Figures 3–5.  Figure 3 displays scatter plots comparing the quantitative MRI measurements of MBF with those obtained from the microsphere analysis. In each panel, the line of unity and a line of best fit are shown for reference. The correlations with microsphere MBF were uncorrected (*y* = 0.47*x* + 1.1, *r* = 0.70), Th1 (*y* = 0.53*x* + 1.0, *r* = 0.71), Th2 (*y* = 0.62*x* + 0.86, *r* = 0.73), Em1 (*y* = 0.82*x* + 0.86, *r* = 0.77), and Em2 (*y* = 0.72*x* + 0.84, *r* = 0.75).  Figure 4 displays Bland-Altman plots for the data shown in Figure 3. Compared to microsphere MBF, the mean MBF difference and 95% confidence interval were uncorrected (−0.75 mL/min/g, −3.66 to 2.16 mL/min/g), Th1 (−0.56 mL/min/g, −3.41 to 2.29 mL/min/g), Th2 (−0.46 mL/min/g, −3.26 to 2.34 mL/min/g), Em1 (0.26 mL/min/g, −2.67 to 3.19 mL/min/g), and Em2 (−0.11 mL/min/g, −2.95 to 2.73 mL/min/g), respectively.

Uncorrected MBF measurements were significantly lower than microsphere MBF (*p* = 0.0001) and MBF corrected with both empirical methods (*p* < 0.0001 and *p* = 0.0007 for Em1 and Em2, resp.), but not significantly different from Th1 or Th2 results. Data corrected with the theoretical methods were significantly lower than data corrected with method Em1 (*p* = 0.0001 and *p* = 0.001 for Th1 and Th2, resp.), but not significantly different from the Em2 or microsphere results. Data corrected with the empirical methods were not significantly different from each other or from the microsphere results.

Figure 5 displays mean (± one standard deviation) results from each of the flow quantification methods tested here. The top row corresponds to segments where the microsphere measurement for flow was in the top 50% of the overall data and the bottom row corresponds to the lower 50% of the data. The mean microsphere flow for the lower half of the data was 1.80 mL/min/g. Both uncorrected flow results and results corrected with methods Th1 and Th2 reported mean flow within 10% of the microsphere results. Flow corrected with the empirically derived methods was higher than the microsphere results by 25% and 15% for Em1 and Em2, respectively, though these differences were not significant. For flow values in the upper 50% of the data, the uncorrected data significantly underestimated microsphere flow by an average of 31% (*p* < 0.0001). The theoretical corrections also significantly underestimated microsphere flow by an average of 25% and 19% for Th1 and Th2, respectively (*p* < 0.0001). Flows corrected with the empirical methods were not significantly different from the microsphere flow (1% and 9% differences for Em1 and Em2, resp.).

## 4. Discussion

When using saturation prepared sequences for MR perfusion imaging, the relationship between MR signal and CA concentration will always be nonlinear. In order to obtain more accurate quantitative values, MR signal should first be corrected for this nonlinear saturation prior to applying any mathematical flow models. In this study, we compared four methods for correcting the MR signal in quantitative perfusion experiments and evaluated the results from each method against MBF measurements calculated with fluorescent microspheres in canine models of myocardial perfusion. Th1 has previously been shown to accurately convert SI to CA concentration in CA-doped saline phantoms [22]. Th2 was also validated in phantoms. In volunteers, Th2 corrected MBF values were higher than uncorrected values and fell within published ranges for resting and vasodilated MBF [19]. The present study is the first to compare several signal saturation correction methods against gold standard microsphere MBF.

Our results show that each of the correction methods tested here resulted in improved MBF estimation, as shown by an increased correlation to and smaller mean difference from microsphere results, as compared to uncorrected MRI data. The results presented here suggest that the myocardial signal will be saturated by as much as 32% when the concentration of CA in the myocardial tissue reaches 1.0 mmol/L. If uncorrected, this signal saturation will result in similar underestimation of true MBF. This is illustrated in Figure 5, where quantification of flow from uncorrected signal curves led to an average of 31% underestimation of MBF in the upper half of flow values measured here. Hsu et al. estimated peak myocardial contrast concentration ranged from 0.77 to 1.32 mmol/L for vasodilated first-pass perfusion using a 0.1 mmol/kg bolus in healthy volunteers, and correction of signal saturation using a method similar to Th2 increased MBF by an average of 28.3%. Although we used a lower CA bolus of 0.05 mmol/kg, the average MBF in the top 50% of our data was also higher than the average vasodilated MBF in the Hsu study (5.2 versus 3.8 mL/min/g) resulting in a similar degree of signal saturation.

We note that none of the corrected MR-based MBF measurement methods tested here resulted in significantly different results from the microsphere results, when all of the sector flows are considered. The results shown in Figures 3 and 4 suggest that the impact of signal saturation increases with MBF. This is expected, as regions of high flow will also have the largest concentrations of CA which result in MR signal saturation. When we tested only the largest 50% of the flow values as measured by microspheres, the MBF values from the MR data corrected with methods Th1 and Th2 were significantly lower (*p* = 0.001) than the empirically corrected MR data or microsphere results.

Each of the correction methods we tested here can be applied retrospectively as a look-up table. For the theoretical methods, implementation requires rederiving the model for a specific pulse sequence and acquisition parameters. In this work, we show that the theoretical models can be successfully applied to a turboFLASH sequence (in contrast to echo planar based imaging methods, which were used in the original implementation of each method). The empirical method can also be applied retrospectively, though a specialized imaging session is required for each combination of pulse sequence and acquisition parameters, which may potentially limit its clinical feasibility. The functional form for the empirical correction given in Em2 suggests that future work may focus on a set of experiments to empirically determine the relationship between SI and CA concentration over a range of imaging parameters for a given pulse sequence type (i.e., saturation prepared turboFLASH as used here). This functional form for the relationship would be both less sensitive to measurement noise and more flexible in imaging parameter selection than a single, empirically determined curve.

As mentioned previously, there are important differences between our implementation of Th1 and Th2 and what was used in the original studies. In this work we modified both methods by replacing the signal equation for EPI with the equation for turboFLASH imaging, as this sequence is more commonly employed in contemporary clinical perfusion imaging. Also, we normalized the perfusion images by the precontrast signal intensity as opposed to proton density weighted images as in [19, 22]. This was done to facilitate broader generalizability of the signal correction methods outlined here. Acquisition of proton density weighted images is not part of most standard perfusion imaging protocols. To test the impact of the SI normalization on MBF calculation, we implemented proton density weighted image normalization in the theoretical methods and applied those corrections to the data presented above. No significant differences between the two normalization schemes were found.

As in the previous implementations of the Th1 and Th2 methods, we did not correct for incorrect flip angle or slice profile effects. We also did not measure the saturation efficiency of the saturation preparation in our imaging sequences. Each of these may have contributed to the underestimation of higher flow values seen in our results.

First-pass perfusion MRI is increasingly being utilized for the assessment of myocardial ischemia, and data supporting the qualitative assessment of perfusion MRI for ischemia is now available from large, multicenter trials [3] and registries [31]. Absolute MBF measurement by perfusion MRI has advantages over qualitative assessment [13] but has only been applied in relatively small, single center studies [32]. A major challenge to the broad application of MBF has been the requirement for specialized bolus strategies or pulse sequences to maintain linearity in the blood pool and myocardium. Emerging techniques to estimate the arterial input function without the need for a dual bolus or dual echo strategy [8, 17] combined with the saturation correction methods employed in this study create the possibility for absolute MBF to be measured from almost any first-pass perfusion study.

One potential limitation of this work is that we did not implement any composite or BIR-4 RF pulses, which have been shown to reduce sensitivity to B0 or B1 inhomogeneity [33]. As a result, some of the images may be affected by additional artifact resulting in errors in the flow measurements. In addition, we have also chosen to neglect any *T*_2_ effects in the theoretical correction methods. This was done intentionally to match the originally described methodology as closely as possible and may affect the signal correction for these two methods at high CA concentrations.

The aim of this work was to compare four methods for correcting myocardial signal saturation in saturation recovery perfusion imaging in a canine model of myocardial perfusion. In 15 experimental perfusion imaging studies, the saturation-corrected MRI data provided MBF measurements closer to those given by fluorescent microspheres than uncorrected data, particularly in regions of high flow. Future studies should focus on validating the methods presented here in human data with the aim of applying saturation correction to quantitative stress perfusion studies with clinically viable CA doses.
